# Valorisation of spent mushroom substrate by secondary microbial fermentation

**DOI:** 10.1007/s00253-025-13696-8

**Published:** 2026-01-20

**Authors:** P. W. Baker, R. Bragança, A. J. Lloyd, A. Charlton

**Affiliations:** 1 Biocomposites Centre, Bangor University, Deiniol Road, Bangor, Gwynedd, LL57 2UW Wales UK; 2 Department of Life Sciences, Aberystwyth University, Aberystwyth, Ceredigion, SY23 3DA Wales UK

**Keywords:** Two-step fermentation, Second stage fermentation, *Lentinus*, *Pleurotus*, Hemicellulose, Cellulose

## Abstract

Global mushroom production exceeds 45 million tonnes, involving growth on a lignocellulosic substrate. More than 70% of these mushrooms are considered wild mushrooms that are grown on a lignocellulose substrate. At the end of the growth cycle, the remaining spent mushroom substrate (SMS) is considered waste with little value and is often discarded. The limited information available on the fibre content remaining in SMS indicates that different white-rot fungi can result in significant differences in the hemicellulose and lignin content, which in turn may impact the second fermentation step. Secondary fermentation has found uses in the production of biofuels, as a preserved ruminant feed, for enzyme production, in the formation of microbial compounds, and as an amended fertiliser. Finally, achieving secondary fermentation of SMS could involve the development and use of larger-scale solid-state fermentation. This review article has shown that research into secondary fermentation of SMS has been a relatively new field occurring over the past 14 years.

**Graphical abstract:**

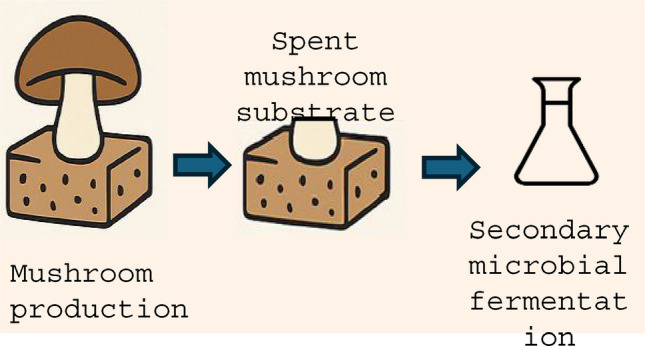

## Introduction

Agricomycetes are fungi that form fruiting bodies and have played an important role as a food source in human society for more than 2000 years, and this use has rapidly developed from Asia into the rest of the world within the last few decades. These fungi are effective in deconstructing the interconnected fibres of cellulose, hemicellulose, and lignin that hold plant cells together. The Agaricomycete family, within the phylum, Basidiomycetes, evolved about 300 million years ago to form white-rot fungi with genes involved in wood decomposition. Some of these genes were lost within members of this family as evolution progressed, forming a non-monophyletic group of fungi (Naranjo‐Ortiz and Gabaldón 2019). This group comprises not only wood saprotrophs, but also a diverse range of fungi acting as plant pathogens, soil and litter decomposers fungi and ectomycorrhizal species.

White-rot fungi are the initial colonisers of parts of the tree, often non-living, where each species of fungus has adapted to grow on particular hardwood tree species. Spores landing on the wood surface germinate and grow into the wood to form a mycelial network. This is achieved using a mixture of lignin-degrading enzymes (laccase, manganese peroxidase, and lignin peroxidase), cellulases, and hemicellulases. The lignin-degrading enzymes are essential for accessing the interconnected layers of cellulose and hemicellulose, yet the fungus derives no energetic gain by degrading this material.

**Fig. 1:**
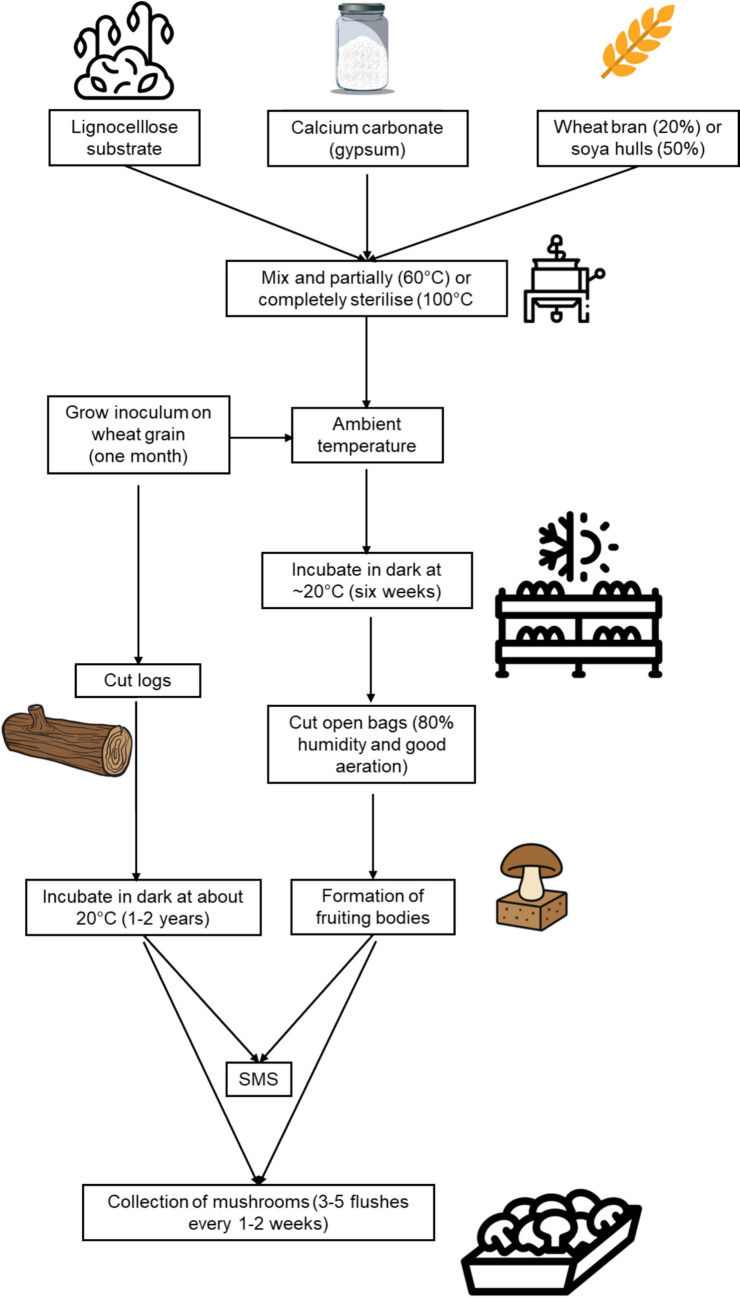
The process for mushroom production

In the mushroom industry, growth of Shiitake mushrooms on cut logs still occurs in large quantities, but this practice has been superseded by growing these fungi on agricultural waste, especially wheat straw, for a significant part of the mushroom industry (Fig. 1). Consequently, different fungal species are grown on highly similar substrates, yet the genetic profile they have developed for growth on various tree species remains.

Much research has been devoted to exploiting these characteristics, such as in the selective lignin degrader *Ceriporiopsis subvermispora,* thereby leaving behind a cellulose-enriched substrate. Other fungi, such as *Trametes versicolor,* can be described as general lignin degraders, resulting in similar levels of hemicellulose, cellulose, and lignin degradation.

The fungi cause a breakdown of the cell walls holding the wood structure together and reduce the fibre content to soluble sugars. It is at this stage, in the natural environment, that faster-growing fungi can enter the decayed wood and begin the second stage of colonisation. However, the decayed material in the mushroom industry is rarely contaminated with other microorganisms, which results in 5 kg of waste being produced for every kg of fresh mushrooms.

In recent years, the natural process of microbial degradation has inspired biotechnological applications, particularly in the valorisation of agricultural residues using co-cultivation (Selegato and Castro-Gamboa 2023). Co-cultivation of two different microorganisms can result in the expression of previously silent genes, such as, in the formation of novel antibiotics arising from defence mechanisms in response to competition. An alternative approach, using sequential fermentation, avoids potential competition between the two microorganisms while leveraging complementary microbial functions for lignocellulose degradation. This process has been successfully commercialised in the production of soy sauce and miso, which both share a similar short primary fermentation with *Aspergillus oryzae*, followed by a much longer secondary fermentation when immersed in brine (Allwood et al. 2021; Devanthi and Gkatzionis 2019). The two methods differ during secondary fermentation due to the presence of different microorganisms and the addition of soybean mash during the production the of miso, whereas nothing is added during the production of soy sauce.

This review has focused on the waste produced from the mushroom industry with a view to secondary fermentation. Articles used in this review were found via the Web of Science search webpage using the terms “spent mushroom substrate” combined with either “two-step fermentation” or “secondary fermentation”. No restriction was placed on the dates of publication, and a reasonable attempt was made to cite all relevant publications using other word searches. Another search was performed using Google Scholar to capture any publications that might have been missed with Web of Science. As far as we are concerned, this is the first review article to discuss the topic of consecutive two-step fermentation. The publications that were found in this search were arranged into different sectors of industry, and an extensive search of global mushroom production was performed.

**Table 1:** List in order of the most cultivated mushrooms and truffles in the world

Scientific name	Common name	Division	Substrate	Global production (tonnes)	Reference
*Agaricus bisporus*	Button mushroom	Ascomycota	Soil casing	13,260,000	Royse (2014)
*Lentinus edodes*	Shiitake	Basidiomycota	SD, WS, PS	12,900,000	Wang et al. (2024)
*Pleurotus* sp.	Oyster mushroom	Basidiomycota	SD, WS, PS	11,934,000	Royse (2014)
*Auricularia heimuer*	Black wood ear	Basidiomycota	SD, WS, PS	7,100,000	Sun et al. (2022)
*Volvariella volvacea*	Straw mushroom	Basidiomycota	PS, banana leaves, CW	330,000	Bao et al. (2013)
*Flammulina filiformis*	Enoki	Basidiomycota	SD, WS, PS	300,000	Lu et al. (2016)
*Hypsizygus marmoreus*	Shimeji mushroom	Basidiomycota	SD with CSH	85,000	Nakamura (2006)
*Hericium erinaceus*	Lion’s mane	Basidiomycota	SD and SH	66,000	Moore and Chiu (2001)
*Boletus aereus*	Boletus	Basidiomycota	Unculturable	60,000	Yun et al. (1997)
*Grifola frondosa*	Hen of the woods	Basidiomycota	SD	39,000	Barreto et al. (2008)
*Morchella* spp.	Morel	Ascomycota	Soil	150	Ajmal et al. (2015)
*Ophiocordyceps sinensis*	Chinese caterpillar fungus	Ascomycota	Grains or liquid medium	100	Shrestha and Bawa (2013)
*Tuber indicum*	Truffle	Ascomycota	Unculturable	50	Podadera-Rivera and Calderón-Vázques (2025)
Sum of estimates				46,008,300	
Official total				48,335,996	Anon (2022)

The *Pleurotus* sp. comprise of *P. ostreatus* (grey oyster), *P. eryngii* (king oyster), *P. citrinopileatus* (yellow oyster)*, P. djamor* (red oyster), *P.s cornucopia* (branching oyster)

*SD* sawdust, *WS* wheat straw, *PD* paddy straw, *CW* corn waste, *CSH* cotton seed hulls

## The mushroom industry and spent mushroom substrate (SMS)

Altogether, there are 14,000 different types of fruiting bodies from white-rot fungi (mushrooms) where the majority are unpalatable but not harmful if consumed (El-Ramady et al. 2022). Only a small proportion, 643 species currently documented, are poisonous (He et al. 2022). Globally, 2,006 different types of mushrooms were identified from reports as being edible, although only 90 of these have been demonstrated to be cultivable (Li et al. 2021). Consequently, the vast majority of these mushrooms are either uncultivable, or there has been no interest in demonstrating that the existing cultivation technique would be effective with these fungi. The appetite for wild mushrooms, in contrast to the button mushroom of the *Agaricus* species, has only recently begun to gain popularity in the Western Hemisphere during the last couple of decades, which contrasts with Asian markets where many of these mushrooms have been readily available for many years.

Consequently, amongst the white rot-fungi that use a solid wood substrate, Shiitake (*Lentinus edodes*), Oyster mushrooms (*Pleurotu*s sp.), and black wood ear (*Auricularia heimuer*) result in the global production of 12.9, 11.9 and 7.1 mega-tonnes of mushrooms, respectively (Table 1). Additionally, some species of white-rot fungi are grown for medicinal purposes, e.g. *Trametes versicolor*, *Ganoderma lucidum,* albeit in small quantities. Growth of all these mushrooms leads to large quantities of SMS that must be managed, which has often involved disposal in landfills or spreading on cultivated agricultural land as a source of nutrients. It is impossible to find data on the quantities of SMS deposed by each country. However, it is generally assumed that 5 kg of SMS remains for every 1 kg of mushrooms produced (Royse, 2014). China is by far the largest producer of mushrooms in the world with 41.1 mega-tonnes followed by Japan with 0.5 mega-tonnes and India as the sixth largest producer with 243 thousand tonnes. The majority of mushrooms in these three countries comprise wild mushrooms grown on a lignocellulose substrate. In contrast, most mushrooms grown in Western countries are button mushrooms on soil casing. Shiitake is the most common wild mushroom produced globally (Table 2) and the quantities of this mushroom production account for a small proportion in Western countries. Shiitake production has shown a significant increase in production by about 10% from the years 2019 to 2022 (USDA 2022) , and it is likely that other countries in the world are showing similar levels of growth.

There are also many potential applications of SMS arising from the commercial production of a range of speciality mushrooms, which have been extensively described in review articles. These include animal feed, fertiliser, bioremediation through absorbing heavy metals, biocomposites for construction applications, energy production, and the recovery of compounds for cosmetics and pharmaceuticals (Antunes et al. 2020; Ma et al. 2025). Another review focused on the extraction of enzymes from SMS of *Pleurotus pulmonarius* for use in the degradation of polycyclic aromatic hydrocarbons, phenolic compounds, recalcitrant fungicides and herbicides, petroleum compounds, and textile dye decolourisation (Phan and Sabaratnam 2012).

**Table 2:** Mushroom production in thousands of tonnes by highest producing nations

Country	Total	Button	Shiitake
China	41,100 ^[1]^	637 ^[4]^	11200 ^[6]^
Japan	469 ^[1]^	0 ^[5]^	88.8 ^[7]^
Poland	379 ^[2]^	380 ^[4]^	1.9 ^[8]^
USA	344 ^[2]^	150 ^[4]^	0.5 ^[9]^
Netherlands	260 ^[2]^	270 ^[4]^	ND
India	243 ^[1]^	50 ^[4]^	ND
Spain	164 ^[2]^	110 ^[4]^	ND
Canada	138 ^[1]^	87 ^[4]^	ND
Russia	111 ^[1]^	172 ^[4]^	ND
France	123 ^[2]^	86 ^[4]^	ND
UK	93 ^[3]^	ND	ND
Germany	84 ^[2]^	100 ^[4]^	ND
Ireland	68 ^[2]^	110 ^[4]^	ND
Italy	68 ^[2]^	ND	ND
Turkey	55 ^[3]^	ND	ND
Hungary	40 ^[2]^	ND	ND
Belgium	28 ^[2]^	ND	ND
Romania	14 ^[2]^	ND	ND
Portugal	13 ^[2]^	ND	ND
Lithuania	9 ^[2]^	ND	ND

*ND* not determined

^[1]^ Bijla and Sharma (2023)

^[2]^Eurostat (2023)

^[3]^Anon (2023)

^[4]^ Van Griensven and Van Roestel (2004)

^[5]^ Kobayashi et al. (2020)

^[6]^Singh et al. (2021)

^[7]^Team (2020)

^[8]^USDA (2022)

Ma et al (2025) highlighted the competition between SMS with traditional lignocellulosic sources that are often in abundant supply, and the large investments that would be required by mushroom companies into diversifying the use of SMS, with a limited return on income. This is further compounded by the significant variation in the composition of SMS due to the range of lignocellulose substrates used during the preparation of the original mushroom substrate and differences in the types of mushrooms being cultivated. Fibre composition of SMS varies with both the original substrate and fungal species (Table 3). Statistical analysis was performed on data that had revealed the extent of variation by determining the theoretical minimum, median, and maximum values to create new data sets. The effect of fungal degradation on cellulose appeared to be insignificant for some types of lignocellulose substrates, such as rubber tree sawdust and paddy straw, but not with cotton seed hulls. In contrast, the effect of fungal degradation on hemicellulose and lignin within each type of lignocellulose substrate was often significant. Fungal decomposition of rubber tree sawdust revealed differences with hemicellulose and lignin, but not with cellulose, between the different fungal species. These results revealed that a higher level of hemicellulose was degraded by *Pleurotus ostreatus* compared with *Auricularia auricula* and *P. pulmonarius*. In contrast, lignin degradation for *P. ostreatus* was lower compared with *A. auricula* and *P. pulmonarius*. Likewise, similar differences were found with the contents of hemicellulose and lignin, but not with cellulose, in SMS of paddy straw degraded by different fungi. It is also important to note that inherent differences in fibre content may occur within different varieties of the same plant species. Furthermore, this is compounded by differences in the choice of additional substrates used in the preparation of mushroom substrate (Fig. 1).

**Table 3:** Fibre composition of SMS in relation to mushroom species and original mushroom substrate

Species	Components of original mushroom substrate	Cellulose	Hemicellulose	Lignin	Reference
*Pleurotus eryngii*	Bagasse 21%, saw dust 21%, bran 18.4%, corncob 18.4%, corn flour 6.7%, cottonseed hull 4.2%, bean pulp 8.4%, CaCO_3_ 1%, lime powder 1%	23.08 ± 0.16 ^b^	26.98 ± 0.47 ^cd^	11.62 ± 0.29 ^bc^	Luo et al. (2018)
*Pleurotus florida*	Corn cob 70%, cottonseed husk 30%, and N and P fertilizers	22.7	18.5	13.6	Leong et al. (2022)
*Lentinus edodes*	Birch sawdust 28%, wheat grain 3.5%, wheat bran 3.5%, CaCO_3_ 0.35%	26.6	31	23.3	Leong et al. (2022)
*Flammulina velutipes*	Cotton seed hulls, wheat bran, sawdust, calcium carbonate and lime	35.0 ± 0.86 ^a^	18.4 ± 0.08 ^cd^	22.7 ± 0.11 ^a^	Wu et al. (2013)
*P. eryngii var. tuoliensis*	Cottonseed hull 40%, corncob 30%, bran 20%, corn flour 8%, lime 1.5%, gypsum 0.5%	22.11 ± 0.51 ^ab^	25.75 ± 0.45 ^cd^	9.54 ± 0.14 ^ab^	Luo et al. (2018)
*Flammulina velutipes*	Rice bran 43.38%, corncob 36.99%, bran 10.96%, beet residue 6.85%, CaCO_3_ 0.91%, and quicklime 0.91%	16.97 ± 0.34 ^a^	28.48 ± 0.86 ^de^	7.95 ± 0.14 ^a^	Luo et al. (2018)
*Auricularia auricula*	Rubber tree sawdust	33.45 ± 0.25 ^c^	29.40 ± 1.90 ^d^	19.56 ± 0.18 ^d^	Supmeeprom et al. (2024)
*Pleurotus ostreatus*	Rubber tree sawdust	32.38 ± 0.08 ^c^	25.02 ± 0.07 ^c^	22.75 ± 0.01 ^f^	Supmeeprom et al. (2024)
*Pleurotus pulmonarius*	Rubber tree sawdust	31.92 ± 1.06 ^c^	27.78 ± 1.32 ^cd^	19.95 ± 0.05 ^d^	Supmeeprom et al. (2024)
*Pleurotus florida*	Wheat straw	37.5 ± 1.1 ^cd^	18.6 ± 0.5 ^a^	20.5 ± 1.2 ^de^	Rajavat et al. (2020)
*Pleurotus ostreatus*	Wheat straw	30.5 ± 0.3 ^c^	17.8 ± 0.5 ^a^	19.4 ± 0.4 ^d^	Fang et al. (2020)
*Pleurotus forida*	Wheat and paddy straw	39.47 ± 1.02 ^de^	24.14 ± 1.75 ^bc^	13.63 ± 1.19 ^c^	Devi et al. (2022)
*Volvariella volvacea*	Paddy straw	41.05 ± 1.03 ^e^	20.79 ± 2.39 ^ab^	10.02 ± 1.81 ^c^	Supmeeprom et al. (2024)

Statistics was performed using one-way analysis of variance with the Tukey post hoc test using SPSS version 27. Numbers with the same letter in a column are not significantly different and those with no-matching letters are significantly different (*P* < 0.05). Some data points were not replicated

Previous studies have shown that hemicellulose is an important nutrient source for different white-rot fungi (Baker and Charlton 2023; Baker et al. 2017). The current observation is that different fungal species will have different effects on the final contents of hemicellulose and lignin of the SMS. This will require further studies to investigate the effects of different fungal species on the same type of mushroom substrate. This would have to involve uniformity in the set-up of experiments and larger data sets to provide higher statistical outcomes. White-rot fungi produce many different lignin-degrading enzymes, and the anabolic processes to generate these enzymes may reach a tipping point where catabolism of remaining nutrients cannot meet these demands. Consequently, despite nutrients still being available, it is speculated that the fungus will cease to grow, and within the natural environment the fungus would be superseded by other fungi, such as *Trichoderma* sp., which produce a lower diversity of lignin-degrading enzymes, as well as other microorganisms.

## Secondary fermentation of SMS

**Fig. 2:**
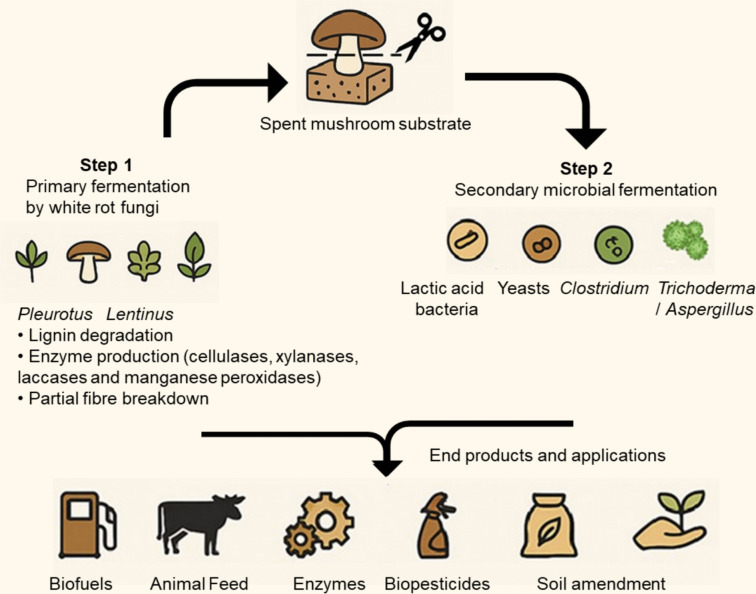
An overview of dual fermentation involving the use of SMS and product formation

A number of studies have investigated the use of another microorganism to continue the degradation process of SMS using solid-state fermentation, or to enable the recovery of useful soluble compounds from the SMS for use in liquid fermentation. In some cases, the secondary microorganism is necessary in order to obtain the desired product, such as in biofuel production, whereas other microorganisms may improve the desired product, e.g. producing higher titres of enzymes than the primary microorganism. An overview of this process is presented in Fig. 2.

## Applications of SMS energy production

One major area involving secondary fermentation is the use of the residual substrate remaining after the harvesting of multiple flushes of mushrooms for bioethanol production, typically with yeasts (Antunes et al. 2020; Ma et al. 2025). The use of enzymes in pre-treating the SMS is preferable to acidic hydrolysis due to the formation of inhibitors that could affect the growth of yeast during bioethanol production (Oguri et al. 2011), although the proportion of easily available sugars might be lower due to fungal activity. More than 90% of the sugars were released from SMS using a cocktail of commercial enzymes at relatively high concentration (e.g. 40 FPU/g-substrate for one of the cellulases from *Acremonium*) when the solid-to-liquid ratio was 5% (w/v), which decreased to 59% with 30% (w/v) solids.

However, the use of commercial enzymes at such high concentrations is expensive, and an alternative strategy to overcome this problem was the use of a microbial consortium on SMS, labelled as SV79, which comprised a stable population of 15 different microorganisms isolated from hot springs in the USA (Zhao et al. 2014). The aim of this study was to produce bioethanol using the consortium, which resulted in 2.13 mM ethanol/g compared with 2.63 mM ethanol/g from *Miscanthus floridulus*. Potentially, our calculations determine that this would equate to 124 L/tonne of SMS. It has been reported that the yields of bioethanol from *P. pulmonarius*-degraded spruce and hardwood substrates were 138 and 92 L/tonne, respectively, following enzymatic hydrolysis (Kousar et al. 2024). The typical yields from first-generation bioethanol production were 447 L/tonne and from second generation using pretreated wheat straw, miscanthus and corn stover were 251, 285, and 330 L/tonne, respectively (Jain and Kumar 2024). The treatments involved ionic liquids, AFEX, or steam explosion, leading to increasing cellulose contents in the remaining substrate. However, the market share of second-generation bioethanol production is marginal compared with first-generation bioethanol produced using corn and sugarcane. This is due to the higher plant assembly costs and higher operational costs associated with second-generation bioethanol production, which often requires government subsidies.

In addition to bioethanol production, fermentation to form 14 g/L butanol with *Clostridium acetobutylicum* from SMS has been reported, (Zhu et al. 2016). SMS was subjected to multiple Organosolv treatments involving 70% ethanol in water at 35 °C, after which the remaining solid was enzymatically hydrolysed for 72 h at 50 °C, and the resulting sugars were recovered as a liquid extract. The development of this approach resulted in the formation of 10 g/L biobutanol with *C. acetobutylicum* on the spent substrate of *P. ostreatus* (Oyster mushroom) (Suresh et al. 2024). This involved a sequential pretreatment with alkali, microwave heating, ultrasonication, and finally enzymatic hydrolysis to achieve almost 93% of the total theoretical butanol yield.

Bioenergy generation can also be achieved through the formation of biogas comprising a mixture of mainly methane and carbon dioxide, or through the production of biohydrogen. Anaerobic fermentation of SMS produces biogas, with yields enhanced by microbial pretreatment using fungi, e.g. *Trichoderma viride* as a fungal inoculant (Zhu et al. 2025) or by allowing spores of *Penicillium* sp. or *Trichoderma* sp. naturally present in the air to colonise the substrate (Pan et al. 2021; Ravlikovsky et al. 2025). The formation of 40 mM biohydrogen by *Clostridium thermocellum* has also been demonstrated on SMS during anaerobic liquid fermentation over 168 h at 55 °C, compared with 17 mM biohydrogen on the material prior to degradation by the mushroom (Hu and Zhu 2017). A further improvement in biohydrogen production to 56 mM was found with the addition of exogenous *β-*glucosidase.

Another aspect into energy generation is the creation of a microbial fuel cell that was filled with SMS, then diluted 100-fold, with distilled water including potassium nitrate as a nitrogen source (Yang et al. 2024). The cell was operated aerobically over four weeks, which resulted in significant denitrification that was accompanied by an average voltage generation of 153 mV. A rich microbial diversity was found around both electrodes, and revealed many types of denitrifying microorganisms around the cathode.

## Food and animal feed production

The SMS can also be used to generate further foods. In one of these processes, the SMS can be combined with fresh mushroom substrate and re-inoculated with another type of mushroom (Dedousi et al. 2024). The optimal mixture level was found to be dependent on the type of substrate being used for the re-cultivation of *P. ostreatus*. *Pleurotus* sp. can be cultivated on a wide variety of lignocellulosic substrates, which may also include re-cultivation on SMS. However, the costs associated with reusing the spent waste may outweigh the relatively low costs of the original lignocellulose substrates (Ma et al. 2025).

The SMS has also been used in probiotic studies to produce ensiled SMS so that it can be effectively stored without the risk of contaminating microorganisms when used as a ruminant feed (Kim et al. 2016). *Lactobacillus plantarum* KU5, originally isolated from spent substrate, was inoculated into the same type of spent substrate consisting of 51% sawdust, 15% beet pulp, 17% corncobs, and 17% cottonseed meal. Ensiling resulted in a decrease from pH 5.3 to pH 3.8, accompanied by an increase in the population of the inoculated bacteria. Acidic conditions were achieved in the 5 L laboratory experiments, and the larger pilot scale experiments of 80 L and one tonne within 7, 14, and 28 days, respectively.

A similar approach was used in another study, but with a different end goal: to form a probiotic liquid suspension for consumption by humans or animals, with the intention of creating useful health-promoting properties to improve gut function gut along with immune-promoting properties associated with the fungal mycelium (Xu et al. 2017). A filtered suspension was obtained from the spent substrate of Enoki mushrooms using heat treatment, which was supplemented with corn syrup and glucose to enable probiotic-type microorganisms to grow. These microorganisms were lactic acid bacteria, acetic acid bacteria, and yeast, which were isolates originally obtained from current fermented foods.

In another study, the SMS was used as a substrate for the production of an essential fatty acid, arachidonic acid, by *Mortierella* sp., and this product would have application as fresh and marine fish feed, leading to normal development and survival (Antimanon et al. 2018). A variety of mixtures were investigated incorporating SMS, rice, soybean, and rice bran. The highest quantities of arachidonic acid (45 mg/g DM) were obtained with SMS and soybean at a ratio of 10:1 which was comparable to yields on a mixture of rice, soybean, and rice bran, indicating that the use of SMS was as effective as the raw materials in producing arachidonic acid.

## Enzyme production

While enzymes can be extracted from the SMS, higher enzyme titres can be recovered by growing species of *Aspergillus* or *Trichoderma,* which grow rapidly and are a rich source of extracellular enzymes. In the natural environment during wood decomposition in forests, these fungi are typically the secondary colonisers. In one study, *Trichoderma reesei* Rut C30, a high cellulase-producing strain, was grown on SMS that had originally contained corn cobs and wheat bran for the cultivation of *Auricularia polytricha* and *nigricans* (both for five generations) and *P. ostreatus* (for three generations) (He et al. 2021). This spent substrate was washed to remove microbial inhibitors, such as furfural and 5-hydroxymethyl furfural, and was supplemented with glucose, urea, and salts to enable fungal growth. Cellulase activities more than eight-fold higher were obtained on the predigested SMS compared with the original material before use in mushroom production. In another study, thermophilic microorganisms were grown at different temperatures of 30°, 50 °C and 70 °C as a consortium from anaerobic digestate on pre-treated SMS with 1% NaOH (Bombardi et al. 2024). SMS was chosen as a cheap substrate that has already been partially digested. Those grown at 50 °C showed improved enzymatic half-lives compared with commercial enzymes operating at the same temperatures. Further work focused on determining specific metabolic profiles for each of the enzymes and genetic analysis.

## Production of microbially useful compounds

The SMS of *Pleurotus eryngii* in combination with the faeces of *Tenebrio molitor* at a ratio of 4:1 was used to grow another inedible mushroom, *Agrocybe chaxingu* (Zeng et al. 2017). This mushroom has potential in the treatment of diabetes and cancer. Growth only on SMS alone, and on SMS with 20% faeces, revealed a biological efficiency of 40% and 63%, respectively. Growth of *Streptomyces albulus* on the SMS of pearl oyster mushroom (*P. ostreatus*), which was amended with either corn syrup or glycerol, resulted in the production of antibacterial compounds, poly(ε-L-lysine) and poly (L-diaminopropionic acid) (Wang and Rong 2022). These compounds were found to have antimicrobial properties and could extend the shelf-life of grape juice by up to 30 days compared with the control without these antimicrobial compounds. In another study, the growth of *Bacillus thuringiensis* on acid hydrolysate from SMS containing free sugars was used as a biopesticide (Wu et al. 2013). However, a low spore density of 10^6^ spores per ml was obtained, and it was suggested that a higher spore density could be achieved with the addition of a nitrogen source.

One white rot fungus, *Lentinus crinitus,* has not yet reached commercial production, as development to achieve this is continuing*. L. crinitus*, a wild South American mushroom, was grown in the laboratory, and the resulting SMS was retained after the fruiting bodies had been collected. Hydrolysates recovered from SMS of this fungus had a positive impact on the growth of *Lactobacillus paracasei* by 55% compared with growth on the undegraded substrate (Dávila et al. 2023). The population density was determined using a microplate reader at a wavelength of 600 nm to measure McFarland units. In addition to the potential probiotic value of *L. paracasei*, antimicrobial compounds associated with *L. crinitus* were assessed against four species of fungi (*Fusarium* sp., *Penicillium* sp., *Rhizopus oryzae*, and *Aspergillus niger*) and four species of bacteria (*Escherichia coli*, *Staphylococcus aureus*, *Bacillus cereus*, and *Salmonella typhimurium*). The best antimicrobial activities were observed against *R. oryzae* and *S. typhimurium* with the hydrolysed extracts at the highest concentration, which was comparable to the antifungal compound ketoconazole and the antibacterial compound oxytetracycline, both at 2 g L^−1^.

In another study, SMS was treated with 6% (w/w) sulphuric acid for 2 h at 120 °C and washed repeatedly until neutralized (Qiao et al. 2011). The remaining SMS was treated with cellulase and xylanase for 72 h at 40 °C. This process released 348 g sugars from each kg of SMS which was sufficient for the growth of *Lactobacillis lactis*. The intention of using this microorganism was in the production of nisin or lactic acid.

## Agricultural uses

SMS can be applied directly to agricultural soil, thereby stimulating the growth of beneficial bacteria and inhibiting crop pathogens such as *Fusarium oxysporium*, *Pythium aphanidermatum* and *Phytophthora capsici*, which may infect tomatoes, cucumbers, and peppers (Mwangi et al. 2024). The value in applying SMS in agriculture can be further increased to provide phosphates, which can be achieved by using the SMS along with additional sulphate and nitrate for the growth of a yeast *Pichia farinose* FL7 (Zhu et al. 2012). This isolate was recovered from a compost heap and was shown to release phosphate across wide ranges of temperature (5–45 °C), pH (3–10) and salinity (0–23% (w/v) NaCl), conditions representative of many soil types. Another study recovered thermotolerant microorganisms from SMS that exhibited higher cellulase activities and could be re-inoculated into SMS as a consortium to improve the fertiliser properties of the SMS (Nguyen et al. 2025). Among the microorganisms was a *Bacillus* sp. that could solubilise inorganic phosphorus. Another similar study except using composting, revealed an increase in *Actinobacteria* and *Ascomycota* that accompanied significant increases in nitrogen and phosphorus contents (Ying et al. 2025). In another study, SMS was inoculated with specific microorganisms and enzymes in a response surface design which evaluated the effect of each variable (Zhang et al. 2024). Analysis of variance revealed that *Bacillus* sp. had some influence in degrading 23% of the hemicelluloses and celluloses, but time and temperature were more important factors during the process. The release of more sugars from SMS could have applications as an animal feed, although this was not explored further.

## Large scale fermentation

There is a need for more research into large-scale solid fermentation to understand potential pitfalls that could hinder the second-stage fermentation process, if applied at industrial scale. There are four different fermentation systems: the drum reactor, the fluidised bed reactor, the packed bed reactor, and the tray reactor (Gómez‐Ramos et al. 2025). The important factors in large-scale fermentation are sufficient aeration to allow continuous growth and the transfer of heat away from the fermentation to prevent overheating that would otherwise inhibit growth.

Within the context of this review, the SMS could be combined to form a large biomass and fermented during the second stage. One of the earliest descriptions of large-scale solid-state fermentation involved 25 L and 3400 L cylindrical bioreactors for the growth of *L. edodes* on wheat straw (Giovannozzi-Sermanni et al. 1997). The results revealed that rotation at 8 revolutions per day was optimal for growth, and higher rotation rates reduced survival of the fungal mycelium. Survival may be reduced without mixing due to oxygen limitation in material furthest from the surface, whereas high rotation speeds may disrupt the mycelium, reducing its viability (Zhao et al. 2014). The knowledge obtained with *L. edodes* could be applied to *P. ostreatus* which has been demonstrated to effectively grow on SMS (Dedousi et al., 2024).

However, the formation of clumps was another technical issue to overcome in another study to produce laccase by *Trametes hirsuta* in a 6 L stirred bioreactor containing an equal mixture of pine wood chips and orange peel (Böhmer et al. 2011). The stirred bioreactor can process large quantities, relying on mixing to redistribute nutrients and the microorganism to ensure equal colonisation throughout the biomass. This type of bioreactor may be beneficial for rapidly growing fungi that sporulate profusely.

Our studies with 1 kg of lignocellulose material, such as *Trichoderma reesei,* in a 3 L solid-state fermentation system (Cleaver Scientific, UK) revealed that growth occurred more rapidly than in smaller 100 g static microcosms (unpublished). This is likely attributable to forced aeration leading to accelerated growth. Spores produced after several days of growth can be redistributed to previously uncolonised areas once stirring is initiated. In all of these experiments, a significant loss in moisture content was observed, presumably as a result of forced aeration, which could affect fungal growth if the levels became too low.

Another description of a large-scale fermentation involved the use of tray reactors containing broken rice with a capacity of 10 L, used with other fungi in pigment production by *Monascus purpurea* (Chaudhary et al. 2023) and *Metarhizium anisopliae* for use as a bio-insecticide (Dallastra et al. 2023). The tray bioreactor is a static system where a thin layer of lignocellulose material allows diffusion of oxygen into the lowest section. This type of bioreactor may be beneficial to microorganisms, e.g. *Trametes versicolor*, that have been found to aggregate into clumps that cannot be easily dispersed in a stirred reactor.

## Conclusion

White-rot fungi are important in the partial degradation of hemicellulose, cellulose and lignin. However, further work is necessary to establish the effects of different white-rot fungi on mushroom substrate that has been prepared using the same lignocellulose substrate. These effects should not only evaluate fibre composition, but also other metabolizable and inhibitory compounds. This would provide a better understanding of the final composition of SMS and how it may be effectively used by the secondary microorganism. It is estimated that globally 164 mega-tonnes of SMS is produced annually which currently has limited secondary income. Genuine commercial opportunities exist involving secondary fermentation of SMS to produce biofuels, as a preserved animal feed, for enzyme production and as an amended fertiliser.

## Data Availability

Data will be made available upon request.
