# Correction: Valorisation of spent mushroom substrate by secondary microbial fermentation

**DOI:** 10.1007/s00253-026-13746-9

**Published:** 2026-02-23

**Authors:** P. W. Baker, R. Bragança, A. J. Lloyd, A. Charlton

**Affiliations:** 1 Biocomposites Centre, Bangor University, Deiniol Road, Bangor, Wales, Gwynedd LL57 2UW UK; 2 Department of Life Sciences, Aberystwyth University, Aberystwyth, Wales, Ceredigion SY23 3DA UK

**Correction: Applied Microbiology and Biotechnology**

10.1007/s00253-025-13696-8

In Table 2 of this article, the fourth column and its corresponding data were inadvertently omitted. Please find the corrected version of the table below, which includes the complete and accurate information.

**Table 2:** Mushroom production in thousands of tonnes by highest producing nations

Country	Total	Button	Shiitake
China	41100 ^[1]^	637 ^[4]^	11200 ^[6]^
Japan	469 ^[1]^	0 ^[5]^	88.8 ^[7]^
Poland	379 ^[2]^	380 ^[4]^	1.9 ^[8]^
USA	344 ^[2]^	150 ^[4]^	0.5 ^[9]^
Netherlands	260 ^[2]^	270 ^[4]^	ND
India	243 ^[1]^	50 ^[4]^	ND
Spain	164 ^[2]^	110 ^[4]^	ND
Canada	138 ^[1]^	87 ^[4]^	ND
Russia	111 ^[1]^	172 ^[4]^	ND
France	123 ^[2]^	86 ^[4]^	ND
United Kingdom	93 ^[3]^	ND	ND
Germany	84 ^[2]^	100 ^[4]^	ND
Ireland	68 ^[2]^	110 ^[4]^	ND
Italy	68 ^[2]^	ND	ND
Turkey	55 ^[3]^	ND	ND
Hungary	40 ^[2]^	ND	ND
Belgium	28 ^[2]^	ND	ND
Romania	14 ^[2]^	ND	ND
Portugal	13 ^[2]^	ND	ND
Lithuania	9 ^[2]^	ND	ND

^[1]^ Bijla and Sharma, 2023

^[2]^ Eurostat, 2023

^[3]^ Anon, 2023

^[4]^ Van Griensven et al., 2004

^[5]^ Kobayashi et al., 2020

^[6]^ Singh et al., 2021

^[7]^ Team, 2020

^[8]^ USDA, 2022

ND Not determined

The original article has been corrected.

